# Editorial on “Use of combined chemotherapy and immunotherapy improves pulmonary arterial hypertension”

**DOI:** 10.1002/pul2.12444

**Published:** 2024-09-20

**Authors:** Lindsay M. Forbes

**Affiliations:** ^1^ Division of Pulmonary Sciences and Critical Care Medicine University of Colorado Anschutz Medical Center Aurora Colorado USA

**Abbreviations**
PAH: pulmonary arterial hypertension,
PVOD: pulmonary veno‐occlusive disease

Recommended therapies for pulmonary arterial hypertension (PAH) have until recently targeted pathways with predominant effects of pulmonary vasodilation.^1^ The recent addition to treatment options of sotatercept, an activin signaling inhibitor which rebalances pro‐ and antiproliferative signaling, bolsters a longstanding goal of targeting pathogenic pulmonary vascular remodeling.^1^, ^2^ Additional remodeling targets exist within the “cancer paradigm” of PAH pathogenesis, i.e. within a variety of proliferative and apoptotic pathways active in pulmonary arterial smooth muscle and endothelial cells.^3^ A potential role for targeting pathways shared with cancer biology is of great interest.

In this issue of *Pulmonary Circulation*, Reddy et al. present the case of a patient with PAH associated with connective tissue disease on triple vasodilator therapy who years into her PAH course received chemotherapy and immunotherapy for metastatic lung adenocarcinoma. In addition to achieving remission of her lung cancer, while treated with chemotherapy and immunotherapy she demonstrated improvement in functional class and REVEAL Lite 2 risk score with overall stable echocardiographic right ventricular function. However, following discontinuation of chemotherapy and immunotherapy she experienced clinical deterioration from PAH with worsening over approximately 21 months of her functional class, risk score, and hemodynamics. Upon her death from acute‐on‐chronic right heart failure, autopsy suggested pulmonary venous fibrous occlusion consistent with pulmonary veno‐occlusive disease (PVOD).

The report is notable for providing a clinical case correlate to a question of great interest, namely whether antineoplastic and/or immunomodulatory agents can improve PAH disease course. The manuscript is particularly strong in providing robust longitudinal data to detail this particular patient's clinical course, including functional assessment, hemodynamics, and even tissue pathology. Naturally, the case is limited in providing generalizable evidence to support a role for antineoplastic and immunomodulatory therapy due to its nature as a case report. Inherent to its presentation of a single case, it is impossible to separate confounding effects from meaningful ones. For example, whether chemotherapy and immunotherapy improved the patient's functional status through an effect on PAH or simply through its positive effect on her malignancy cannot be known. (Notably, her risk score improvement may likewise be attributed to improvement in her functional class and 6‐min walk distance.) Additionally, it is not clear how to reconcile the autopsy diagnosis of PVOD. While pulmonary venous remodeling may be observed in end‐stage PAH,^4^ alternative explanations that this was a longstanding diagnosis or that the chemotherapy and immunotherapy played a role in its emergence are impossible to determine.

Nevertheless, this report provides a rare and well‐documented perspective into the possible effects of antineoplastic and immunomodulatory therapy among patients with PAH. In particular, it is notable that in this particular patient there was no evidence of harm to her comorbid PAH during her active cancer therapy. In fact, despite a complex medical regimen with numerous agents known to pose risks of toxicity, her functional status improved in parallel with her underlying disease states. Ultimately, ongoing and future investigations are needed to determine whether antineoplastic and immunomodulatory therapy may benefit patients with PAH.

## AUTHOR CONTRIBUTIONS

Lindsay M. Forbes conceived and wrote the manuscript.

## CONFLICT OF INTEREST STATEMENT

The author declares no conflict of interest.

## ETHICS STATEMENT

This work does not involve human subjects, animal experimentation, or cell lines.
